# Green Extraction Strategies for Sea Urchin Waste Valorization

**DOI:** 10.3389/fnut.2021.730747

**Published:** 2021-09-13

**Authors:** Stefania Marzorati, Giordana Martinelli, Michela Sugni, Luisella Verotta

**Affiliations:** Department of Environmental Science and Policy, Università degli Studi di Milano, Milan, Italy

**Keywords:** sea urchin's waste, biomass valorization, green extraction, supercritical CO_2_, antioxidants, polyhydroxylated naphtoquinones

## Abstract

Commonly known as “purple sea urchin,” *Paracentrotus lividus* occurs in the Mediterranean Sea and the eastern Atlantic Ocean. This species is a highly appreciated food resource and Italy is the main consumer among the European countries. Gonads are the edible part of the animal but they represent only a small fraction (10–30%) of the entire sea urchin mass, therefore, the majority ends up as waste. Recently, an innovative methodology was successfully developed to obtain high-value collagen from sea urchin by-products to be used for tissue engineering. However, tissues used for the collagen extraction are still a small portion of the sea urchin waste (<20%) and the remaining part, mainly the carbonate-rich test and spines, are discarded. Residual cell tissues, tests, and spines contain polyunsaturated fatty acids, carotenoids, and a class of small polyphenols, called polyhydroxynaphthoquinones (PHNQ). PHNQ, due to their polyhydroxylated quinonoid nature, show remarkable pharmacologic effects, and have high economic significance and widespread application in several cosmetic and pharmaceuticals applications. A green extraction strategy aimed to obtain compounds of interest from the wastes of sea urchins was developed. The core strategy was the supercritical CO_2_ technique, characterized by low environmental impacts. Fatty acids and carotenoids were successfully and selectively extracted and identified depending on the physical parameters of the supercritical CO_2_ extraction. Finally, the exhausted powder was extracted by solvent-based procedures to yield PHNQ. The presence of Spinochrome A and Spinochrome B was confirmed and extracts were characterized by a remarkably high antioxidant activity, measured through the 2,2′-azino-bis(3-ethylbenzothiazoline-6-sulfonic acid) (ABTS) assay. Overall, the selective and successive extraction methods were validated for the valorization of waste from sea urchins, demonstrating the feasibility of the techniques targeting added-value compounds.

## Introduction

Food waste valorization implies its conversion into higher-value products that contribute back to the supply chain or other kinds of applications that support the circular economy approach where useful materials, once seen as waste or by-products, are recycled. Given the high interest in the environmental issues, already remarked by the 17 Sustainable and Development Goals of Agenda 2030, where sustainability is the core, nowadays a lot of research is ongoing in the field of food industrial sustainability during the production process and at the end of the chain (1). This points to ensure zero-waste strategies since both by-products and end-products are granted a second life. The shift from a linear economy model to a circular one differs substantially in the perspective on sustainability, aiming to ensure healthy and safe living while causing less harm to the environment.

Within this context, sea urchins, belonging to the class Echinoidea of the echinoderm phylum, are a highly appreciated food resource. Indeed, around 75,000 tons of sea urchins are sold annually worldwide for gonads consumption and in recent decades, the demand for sea urchins is increasing (2, 3). Italy is the main consumer among the European countries with 30 million sea urchins annually consumed only in Sardinia (4). However, since the yellow-orange gonads are the only edible part, representing a small fraction of the entire animal, the remaining ends up as waste. Recently, an innovative methodology was successfully developed to obtain high-value collagen from sea urchin by-product tissues to be used for regenerative medicine applications (5). Prototypes of biodegradable medical devices made of this eco-friendly marine collagen were developed and characterized, analyzing their microstructure, mechanical performances, *in vitro* cytocompatibility, and, recently, also their regeneration efficiency in *in vivo* animal model (6–8). However, the tissues used for the collagen extraction are a small portion of sea urchin waste (<20%), and the remaining part, mainly the carbonate-rich test and spines, would be discarded again.

The interest in this secondary waste, comprising both the residual tissues and tests and spines, is however still high, considering that marine organisms, such as sea urchins, have been widely studied for their richness in secondary metabolites, displaying potent activities and exciting pharmacological potential. Examples abound of antitumor, antiviral, immunosuppressive and antimicrobial agents, and cardiac stimulants (9). Several studies have indicated that the gonads of sea urchins are rich in important bioactive, such as polyunsaturated fatty acids, sterols, and carotenoids. An extract of gonad tissue has also revealed effective anti-inflammatory and anti-diabetic properties (10). Even more interesting, a class of small polyphenols characterized by a hydroxylated quinonoid chemical structure, identified as polyhydroxynapthoquinones (PHNQ), has been described, exhibiting a wide range of biological activities. More than 40 naphthoquinones have already been identified from different sea urchin species, Echinochrome A and the Spinochromes A-E are the best-known (11). They are generally derivatives of polyhydroxy-1,4-naphthoquinones, substituted with ethyl, acetyl, methoxy, or amino groups (12, 13). In addition to the presence of a chromophore that is responsible for the PHNQ characteristic colors, pigments have high economic significance because of their widespread applications as antibacterial and fungicidal agents. Furthermore, they have shown protection against UV-induced damage and their ability to scavenge the reactive oxygen species (ROS) pointed to their use as antioxidants or as an immune modulator (14, 15). Particularly, Echinochrome A was introduced as an active substance of the drug Histochrome, commonly used in ophtalmological and cardiological clinical practice in Russia (15).

Considering the high potential of the compounds present in sea urchins, this study aimed to develop extraction strategies resulting in the downstream processes able to exhaust the added value from this food waste. First, targeting the lipid content, a “green” extraction was developed, by means of supercritical carbon dioxide. This technology, employing CO_2_ as an extraction fluid is indeed characterized by low environmental impacts: organic solvents are avoided ensuring safe and selective processes directly on the powder without any pretreatment, with the possibility to recycle the employed CO_2_ in the industrial plants. In a second stage, a small percentage of ethanol, acting as a polarity modifier, was employed together with supercritical CO_2_ to target more polar compounds.

The absence of any excessive heating or destructive step allowed a further and final extraction, able to successfully yield PHNQ. The pigments were selectively and separately obtained from the previously extracted powder, their chemical structure was assigned, and their antioxidant activity was finally assessed.

## Materials and Methods

### Chemicals

All chemicals were used without further treatment. Acetonitrile, methanol, and water, when used for liquid chromatography, were purchased from Merck (Germany) as ultra-performance liquid chromatography-grade. Anhydrous sodium sulfate, sodium carbonate, formic acid, ethanol, ethyl acetate, 2,2′-azino-bis(3-ethylbenzothiazoline-6-sulfonic acid) (≥ 98%; ABTS), Folin–Ciocalteu reagent (2M), and 6-hydroxy-2,5,7,8-tetramethylchroman-2-carboxylic acid (97%; Trolox®) were purchased from Merck (Germany). Gallic acid was purchased from Carbosynth (UK), astaxanthin, and lutein (90%) from Acros Organics (Thermo Fisher, Germany), and β-carotene (99%) from Alfa Aesar (Thermo Fisher, Germany). The silylation reagent (SILYL-991) was purchased from Macherey-Nagel (Germany).

### Starting Material

In the study, 50 kg of wastes of sea urchins, belonging to the species identified as *Paracentrotus lividus* (origin: Adriatic Sea) were kindly donated from the restaurants close to the University of Milan after gonads removal and kept at −20°C. The recovered material was then lyophilized to eliminate the residual water (between 50 and 60% by weight of starting biomass) and better preserve the material. Lyophilization was conducted in 24 h using a 5Pascal Srl (Italy) freeze dryer equipment. The lyophilized material was then ground using a knife mill (Fritsch, Pulverisette 11, Italy), at 10,000 rpm for 20 s. To avoid powder heating during blending and the consequent degradation of thermolabile species, liquid nitrogen was added.

### Supercritical Fluid Extractions

Supercritical fluid extractions (sc-CO_2_) were performed using a pilot unit SFT110XW System supplied by Supercritical Fluid Technologies, Inc. (DE, USA). It consisted of a 100 cm^3^ stainless steel extraction vessel inserted in an oven, a constant pressure piston pump (SFT-Nex10 SCF Pump) with a Peltier Cooler, a 515 HPLC pump (Waters, Milford, MA, USA) for the co-solvent addition, and a collection vial.

The extraction vessel was filled with a weighted amount of lyophilized and grounded sea urchins waste (about 60 g of powder was used in each trial). The system was sealed, and the oven and restrictor block temperatures were set at 60 and 75°C, respectively. After a literature overview and optimization trials, the operative pressure was set as 200 bar in all experiments. Extractions were performed in triplicate.

Nine cycles, comprising 10 min of maceration time in static conditions and 30 min of dynamic conditions, were performed for the extraction experiment. In dynamic conditions, the valves were opened, and the extract was collected in a vial, keeping the CO_2_ gas at a constant flow rate of 15 SCFH (standard cubic feet per hour). The extracts were then stored in a freezer for subsequent analyses.

To further extract the material, shifting the window of extractable compounds toward more polar targets, a subsequent extraction was performed on the same material using sc-CO_2_ added with 10% of ethanol as co-solvent. 4.6 ml of ethanol was loaded into the vessel before pressurization. Once the set pressure (200 bar) was reached and a static period of 10 min was maintained, valves were opened to collect the sample for 30 min in dynamic conditions. During the dynamic extraction, the first four cycles were performed using an ethanol flow rate of 0.5 ml min^−1^, whereas the last three cycles were carried out with an ethanol flow rate of 1.5 ml min^−1^. The vessel was then depressurized, and the residual biomass was collected. The sc-CO_2_ extraction, in the presence of co-solvent, was run three times for sake of data reliability.

### PHNQ Extraction

The PHNQ extraction from sc-CO_2_ extracted powder was carried out with modifications of the literature method by Powell et al. (16). Briefly, 140 ml of 6M formic acid were added drop-wise under stirring (at room temperature) to 20 g of sea urchin powder, exactly weighted, to decompose the carbonate matrix. The mixture was stirred for 1 h. The suspension was centrifuged at 6,000 rpm for 5 min, and the supernatant was further filtered under vacuum using the Büchner funnel. The acid aqueous extract was then counter-extracted with aliquots of 50 ml of ethyl acetate. The orange/pink organic layers were separated, and the extraction process was repeated three times with fresh aliquots of ethyl acetate. Organic phases were collected and then washed repetitively with milli-Q water to remove the residual formic acid and inorganic salts. This step was repeated until the aqueous phase conductivity and pH were close to the milli-Q water ones. Approximately 10 washing cycles were necessary. Anhydrous sodium sulfate was added to remove the residual water in the organic phase. The extract was then dried by a rotary evaporator (37°C) and finally by a mechanical vacuum pump.

### Total Phenolic Content (TPC)

The total phenolic content was determined using the Folin–Ciocalteu assay. Following a literature method (17), 25 μl of standard solutions of gallic acid (range 0–1 mg ml^−1^) were added with 1.5 ml of deionized water and 125 μl of Folin–Ciocalteu reagent (2M). After 5 min, 0.5 ml of a 15% Na_2_CO_3_ aqueous solution were added. After 2 h incubation in the dark, the absorbance was measured at 765 nm using Jasco V-630 Spectrophotometer (MD, USA). After building the gallic acid calibration line (plotting the gallic acid concentration vs. A_765nm_), the same method was performed on the methanolic solution of PHNQ. Results are expressed as mg _GAE_ g ${}_{\text{PHNQextract}}^{-1}$ (GAE = gallic acid equivalents).

### ABTS Assay

To evaluate the antioxidant activity of the product, the spectrophotometric analysis of ABTS^•+^ radical scavenging activity was performed according to Loganayaki et al. (18). Briefly, a 7 mM ABTS aqueous solution was prepared. Then, ABTS radical cation was produced by reacting ABTS aqueous solution with 2.45 mM ammonium persulfate (final concentration) and allowing the mixture to stand in the dark at room temperature for 12–16 h before use. Prior to the assay, the solution was diluted in ethanol (about 1:75 v/v) to give an absorbance at 734 nm of 0.700 in a 1 cm cuvette. Different concentrations (in the range 0–0.012 mg ml^−1^) of the methanolic solutions of PHNQ extracts were reacted with the ABTS radical cation. The same procedure was carried out using different concentrations of Trolox® solutions. The blank did not contain the sample. After 1 h incubation in the dark, the absorbance was measured at 734 nm. The extract concentration in solution was plotted vs. the percentage of ABTS^•+^ remaining in solution, calculated as in Equation 1:


(1) $$\begin{array}{r} \%\;ABTS^{•+}remaining=\frac{A_{734\;nm,\;1h,\;sample}}{A_{734\;nm,\;1h,\;blank}}\% \end{array}$$

Extract and Trolox® EC_50_ values were read on the graph. Experiments were performed three times.

### Characterization

#### Inductively Coupled Plasma Optical Emission Spectrometry (ICP-OES)

In the study, Ca^2+^ and Mg^2+^ contents in sea urchin waste were quantified by weight using ICP-OES. Before analysis, the powder of sea urchins was decomposed by the treatment with 6 M formic acid. The residual solid was filtered by the Buchner funnel and then by 0.22 μm PTFE syringe filters. The filtered solution was diluted and then analyzed by using Perkin Elmer Optima 8000 equipment (MA, USA). A 5-points calibration line was built for both elements in the range 2.5–20 mg L^−1^; Wavelengths: Ca: 317.933 nm, Mg: 285.213 nm; Plasma: 10 L min^−1^; auxiliary gas: 0.2 L min^−1^; nebulizer (Meinhard K1, Perkin Elmer): 0.55 L min^−1^; peristaltic pump: 1 ml min^−1^.

#### Gas Chromatography-Mass Spectrometry (GC-MS)

Before the GC-MS analyses, samples were subjected to a derivatization process. A modification of a literature method was used (19). Briefly, about 5 mg of each sample were accurately weighed, suspended in 100 μl of anhydrous pyridine, and silylated using 100 μl of N,O-Bis (trimethylsilyl)trifluoroacetamide (BSTFA) containing 1% of Trimethylsilyl chloride (TMCS). The samples were incubated at 60°C and stirred for 2 h. At the end of the reaction, 0.8 ml of ethyl acetate was added. The samples were then filtered (0.22 μm PTFE syringe filters) and analyzed by using an ISQ™ QD Single Quadrupole GC-MS (Thermo Fisher, MA, USA) equipped with a VF-5ms (30 m × 0.25 mm i.d. × 0.25 μm; Agilent Technology, CA, USA). Injection volume: 1 μl, split mode; Oven program: 120°C for 5 min; then 10°C min^−1^ to 200°C; 5 min holding time; then 20°C min^−1^ to 300°C; 15 min holding time; C for 15 min; run time: 38 min. Helium was used as a gas carrier. Ionization mode: electron impact: −70 eV. Acquisition mode: full scan. To identify the chemical structure of the species eluting, the fragmentation pattern of each peak was compared to NIST 2014 database.

#### Ultrahigh-Performance Liquid Chromatography-Tunable Ultraviolet-Electrospray Ionization-Mass Spectrometry (UPLC-TUV-ESI-MS)

The identification and quantification of carotenoids, by comparison to authentic standards (when available) was performed on Acquity UPLC equipment (Waters corp., MA, USA), and the following conditions were used: column: ACQUITY UPLC BEH C_18_ (50 × 2.1 mm, 1.7 um) (Waters, Milford, MA, USA), column temperature: 34°C, and eluents were A: water + 0.1% formic acid and B: acetonitrile + 0.1% formic acid. The flow rate was set at 0.3 ml min^−1^ and the linear gradient elution was: START: 25% A: 75% B; 20 min: 0% A: 100% B; 28 min: 0% A: 100% B; the sample temperature was kept constant at 20°C and the injection volume was 2 μl. Injections have been repeated three times. The detector was an Acquity TUV Detector (Waters, Milford, MA, USA), and the wavelength was set at 445 nm. Data were processed with Empower 3 workstations. Limit of detection (LOD) and limit of quantification (LOQ) were calculated according to Ermer et al. (20). Chromatographic separation was followed by a mass spectrometry (LCQ Fleet Thermofisher, MA, USA) analysis, according to a previous study (21). A positive electrospray mode was used for the ionization of molecules with a capillary voltage of 50 V and at a capillary temperature of 275°C. The heater temperature was set at 40°C, the gas flow rate was 20 (arb), and the spray voltage was 4.0 kV. The monitored mass range was *m/z* 100–900.

#### Ultrahigh Performance Liquid Chromatography-Photodiode Array-Electrospray Ionization-High Resolution Mass Spectrometry (UPLC-PDA-ESI-HRMS)

The identification of the PHNQs in the extract was carried out using UPLC-PDA-ESI-HRMS. The UPLC-PDA-ESI-HRMS analyses were performed by the Unitech COSPECT Mass Spectrometry facility (University of < city>Milan < /city>, Italy). The UPLC equipment was Acquity UPLC I Class (Waters, Milford, MA, USA), and the following conditions were used: column: ACQUITY UPLC BEH C_18_ (50 × 2.1 mm, 1.7 um) (Waters, USA), column temperature: 34°C, and eluents were A: water + 0.1% formic acid and B: acetonitrile + 0.1% formic acid. The flow rate was set at 0.5 ml min^−1^ and the linear gradient elution was set: START: 95% A: 5% B; 5.15 min: 60% A: 40% B; 6 min: 10% A: 90% B; 6.30 min: 10% A: 90% B; 6.60 min: 95% A: 5% B; 10 min: 95% A: 5% B. The sample temperature was kept constant at 20°C and the injection volume was 4 μl. The detector was an Acquity UPLC PDA Detector (Waters, Milford, MA, USA) working in a wavelength range of 210–500 nm. The HRMS detector was a Synapt G2-Si QTof (Waters, USA). The operative parameters are given in the following: ESI Ionization mode, negative ionization polarity, full scan range 50–1,200 *m/z*, leucine enkephalin was the lock mass compound. The used software was MassLynxTM v4.2 (Waters, Milford, MA, USA).

## Results

### Supercritical CO_2_ Extractions

#### Pure Supercritical CO_2_

**Figure 1:**
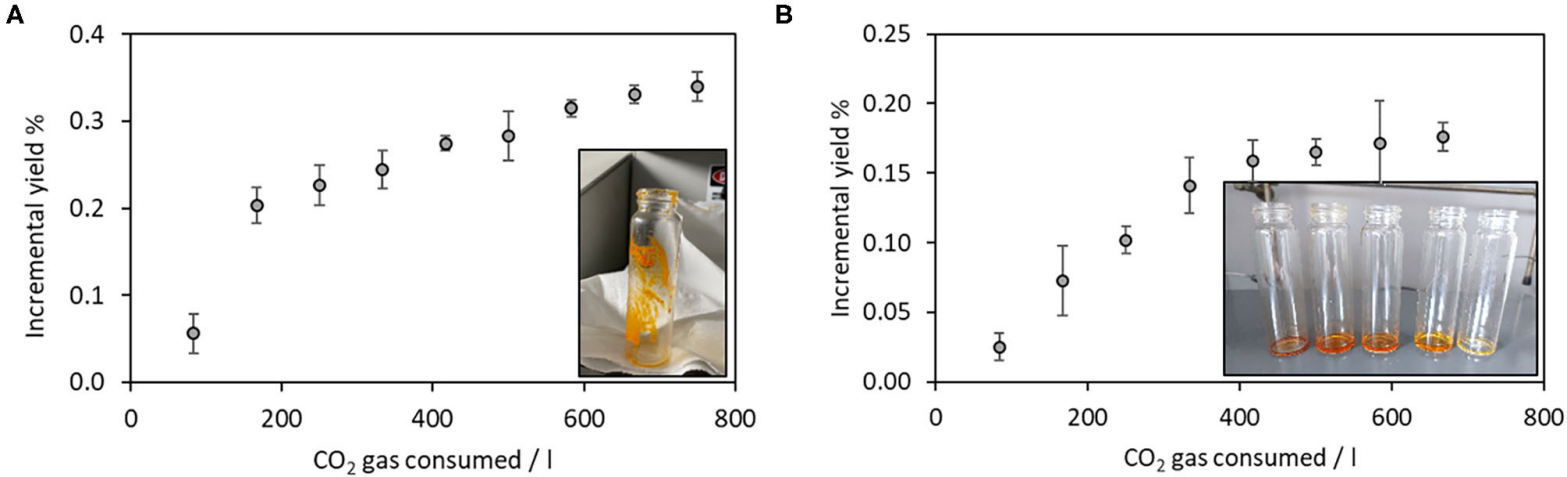
Kinetics of supercritical fluid extraction using **(A)** pure CO_2_; **(B)** using ethanol as co-solvent. Insets show the visual aspect of obtained extracts. Number of replicates = 3.

Figure 1A displays the kinetics of extraction when the supercritical CO_2_ extraction was run on the pristine sea urchin powder. Each point represents the incremental yield (% g _extract_/g _dry biomass_), detected over time and hence over CO_2_ consumption. The alternation of static and dynamic cycles was interrupted when the incremental yield reached a plateau, indicating no more evident extracted mass increment. This ending point was reached after 750 liters of gaseous CO_2_ have been consumed, corresponding to 270 min of extraction in the dynamic conditions. The inset in Figure 1A shows the appearance of the oily extract, characterized by brilliant orange color. The final yield corresponded to 0.34 ± 0.02% by dry weight of the starting biomass.

**Figure 2:**
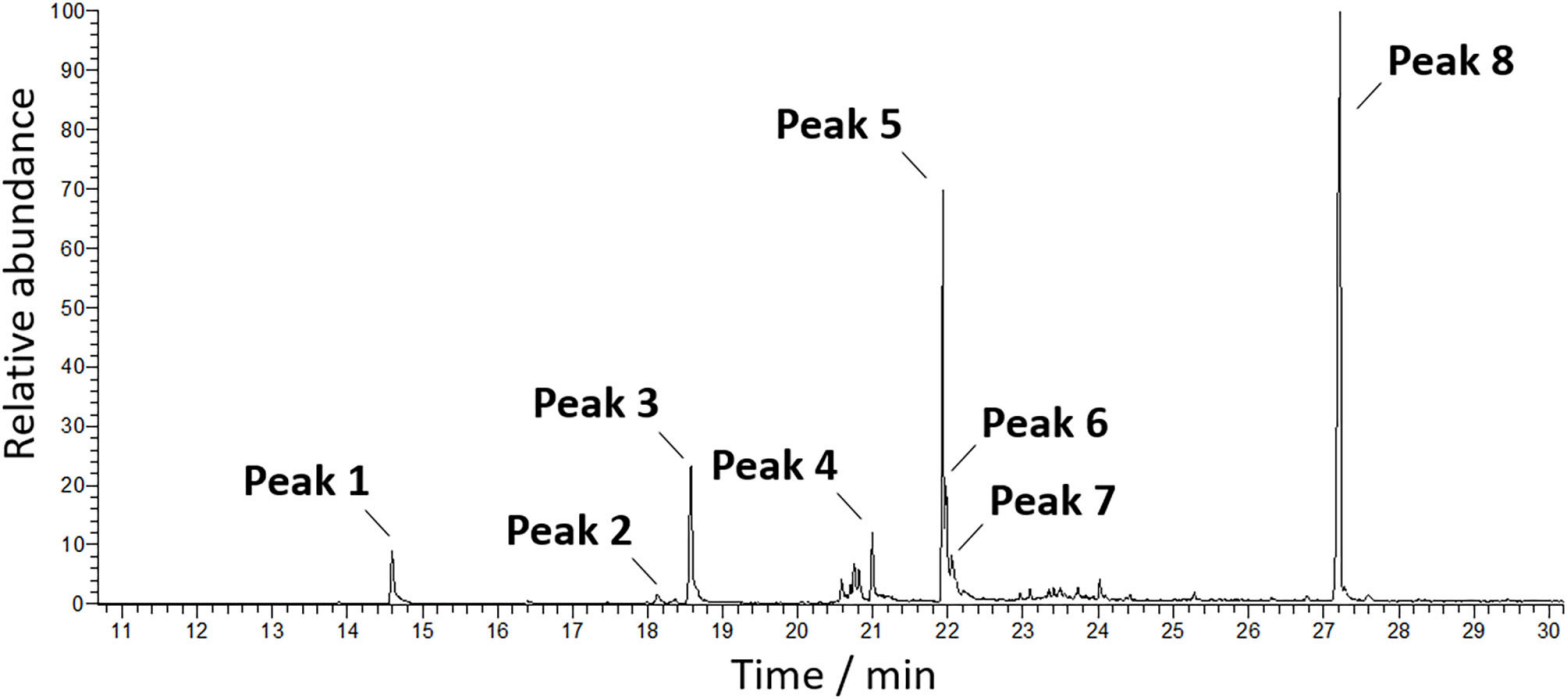
Total ion current-mass spectrometry (TIC-MS) chromatograms of the sc-CO_2_ extract.

The obtained sample was characterized by GC-MS, after BSTFA/TMS derivatization to get the corresponding silyl derivatives. The fatty acid composition is displayed in Table 1 and the GC-MS chromatogram displaying the total ion current is shown in Figure 2. The most intense peak is attributed to cholesterol [peak 8, r.t (retention time) = 27.22 min]. Due to the differences in the fragmentation pattern affecting the ionization current, the relative percentages are given considering only one class of compounds, particularly, the fatty acids. Other species, such as cholesterol, have been identified but not quantified as relative percentages. The most abundant fatty acids, in order of relative abundance, were identified as arachidonic acid and palmitic acid followed by eicosapentaenoic, mirystic, and eicosatrienoic acids.

**Table 1:** Gas chromatography-mass spectrometry (GC-MS) peaks attribution and fatty acids relative abundances.

**Peak**	**Retention time**	**% Area**	**Compound**	**Acronym**
1	14.6	10.0	Myristic acid	14:0
2	18.13	2.2	Palmitoleic acid	16:1 (9c)
3	18.57	24.2	Palmitic acid	16:0
4	20.99	7.4	Stearic acid	18:0
5	21.93	31.2	Arachidonic acid (ω6)	20:4 (5c,8c,11c,14c)
6	21.97	13.9	Eicosapentaenoic acid (ω3)	20:5 (5c,8c,11c,14c,17c)
7	22.05	11.1	Eicosatrienoic acid (ω9)	20:3 (11c,14c,17c)
8	27.22	–	Cholesterol	–
		41.6	SFA	
		56.2	PUFA	

*SFA, saturated fatty acids; PUFA, polyunsaturated fatty acids*.

#### Supercritical CO_2_ + Ethanol as Co-solvent

After the first sc-CO_2_ extraction was run and the extract was collected, a second extraction was carried out on the previously extracted powder, keeping all the physicochemical parameters of the extraction constant but in the presence of ethanol as co-solvent in a 10% with respect to the biomass weight, as a supercritical CO_2_ polarity modifier.

Each point in Figure 1B corresponds to the extract weight after the solvent evaporation by a rotary evaporator (37°C). The plateau was reached in this case again after 750 liters of CO_2_ gas have been consumed, and the extraction trial was stopped. The inset in Figure 1B shows the obtained extracts, corresponding to the first five cycles. The deep orange/red color decreased in the intensity cycle after the cycle, confirming that the biomass was exhausted in its extractable content. The final yield corresponded to 0.18 ± 0.01% by dry weight of the starting biomass.

#### Carotenoids Analysis

The carotenoids identification in the sc-CO_2_ and sc-CO_2_EtOH extracts was performed by liquid chromatography coupled to mass spectrometry (LC-MS).

**Figure 3:**
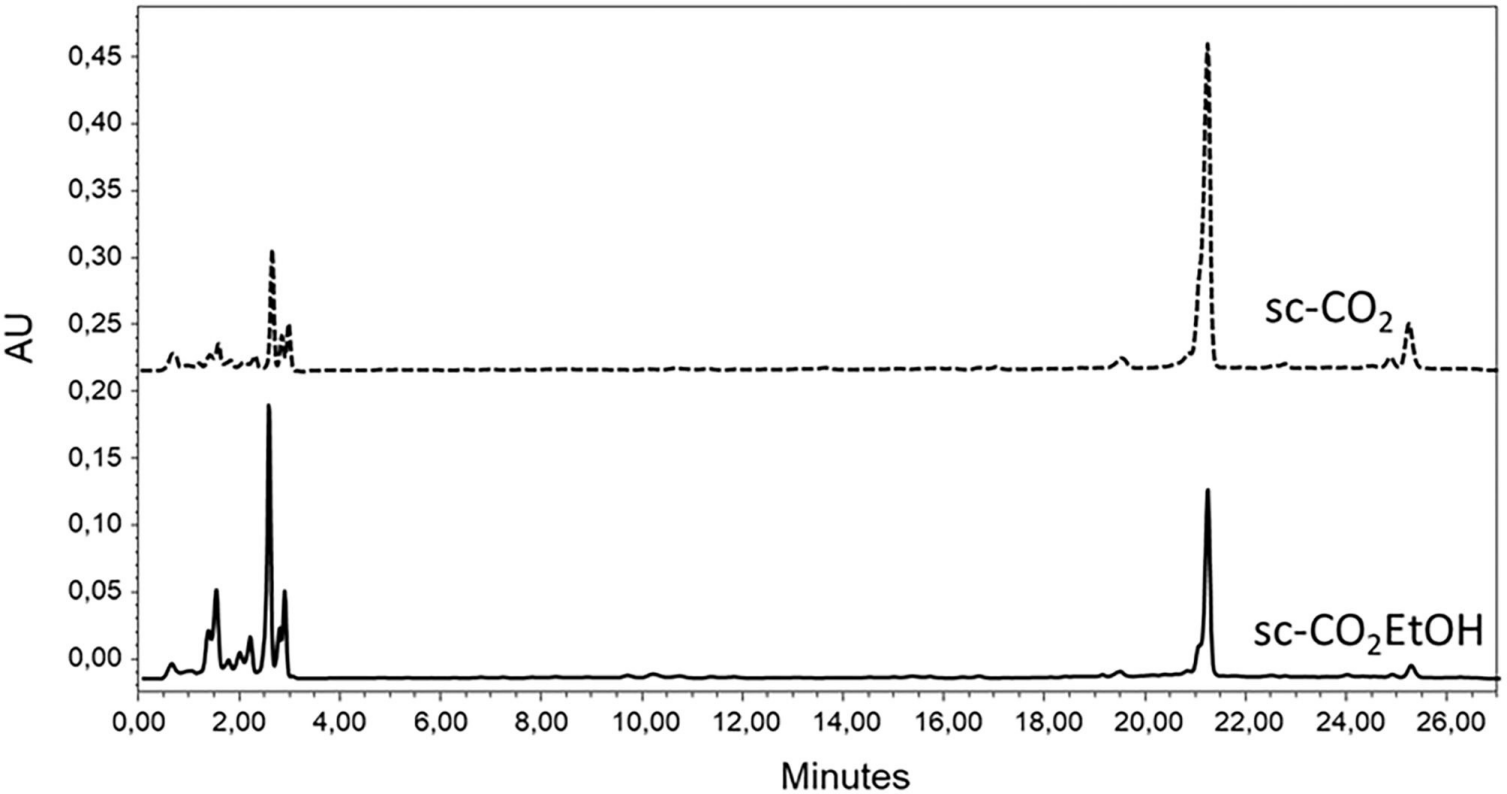
Ultrahigh-performance liquid chromatograms of sc-CO_2_ and sc-CO_2_EtOH extracts (445 nm).

Figure 3 displays the overlap of chromatograms recorded at 445 nm obtained for the sc-CO_2_ and sc-CO_2_EtOH extracts. From mass spectrometry results, it was possible to confirm the presence of astaxanthin, whose peak at a retention time of 2.5 min, was characterized by the main signal at *m/z* = 597.40, corresponding to [M+H]^+^ molecular species and a second peak at *m/z* = 579.40, corresponding to [M-H_2_O+H]^+^ species (MW_astaxanthin_ = 596.84 g mol^−1^) (22, 23). The presence of β-carotene was confirmed by the same strategy, and the peak at t_r_ = 25.3 min was characterized by the main signal at *m/z* = 536.32, corresponding to [M]^+•^, while the secondary signal at *m/z* = 569.04 corresponds to the coordination of methanol molecule (MW_β−carotene_ = 536.87 g mol^−1^). The results are in agreement with the literature data (21, 24–26). By comparison with the authentic standards retention times (t_r_), both the sc-CO_2_ and sc-CO_2_EtOH extracts are found to contain astaxanthin (t_r_ = 2.5 min) and β-carotene (t_r_ = 25.3 min).

Lutein was excluded in the assignments of peaks in the samples chromatograms, as confirmed by the co-injection with lutein authentic standard. The main peak, eluting at 21.2 min was then attributed to echinenone. The presence of echinenone was confirmed by UPLC-TUV-MS. The corresponding peak was characterized by the main signal at *m/z* = 551.44, corresponding to [M+H]^+^ echinenone molecular species (MW_echinenone_ = 550.85 g mol^−1^). The results are in agreement with the literature data (22, 27).

Carotenoids quantification was carried out by UPLC. Six dilutions of the starting standards of astaxanthin, lutein, and β-carotene solutions (in chloroform:acetonitrile = 1:10) were prepared in the range of 0.6–70, 0.9–30, and 0.9–116 μg ml^−1^ for astaxanthin, lutein, and β-carotene, respectively. Standard solutions were injected three times in the UPLC system and monitored at 445 nm. All compounds were eluted as single sharp peaks at a retention time of 2.5 min for astaxanthin, 20.1 min for lutein, and 25.3 min for β-carotene. In the operative concentration range, the trend was linear, with no saturation effects that could bend the linearity. No standards were available for echinenone. Its quantification has been performed using the calibration line of astaxanthin, being the molar extinction coefficients of astaxanthin and echinenone similar [125·10^3^ l and 119·10^3^ l mol^−1^ cm^−1^ (28, 29)]. The area under each peak was quantified by Empower 3 instrumental software and plotted vs. the concentration. The best fit of experimental data in the plot “Peak area vs. [carotenoid]” was, as expected, a straight-line, represented by the following mathematical equations: y = (1.97·10^8^ ± 2·10^6^)x + (-1.2·10^5^ ± 5·10^4^) for astaxanthin standard solutions (LOD = 0.9 μg ml^−1^, LOQ = 2.7 μg ml^−1^), y = (6.81·10^7^ ± 8·10^5^)x + (5·10^5^ ± 2·10^5^) for lutein standard solutions (LOD = 1.1 μg ml^−1^, LOQ = 3.3 μg ml^−1^) and y = (6.76·10^7^ ± 5·10^5^)x + (5·10^5^ ± 1·10^5^) for β-carotene standard solutions (LOD = 1.8 μg ml^−1^, LOQ = 5.6 μg ml^−1^). Linearity was assessed through the evaluation of the coefficient of determination, which should be >0.998; in all cases, it was equal to 0.999.

Based on these results, the calculated equations of the regression line were then employed to determine the carotenoids concentration in the sc-CO_2_ and sc-CO_2_EtOH extracts. Results are displayed in Table 2.

**Table 2:** Carotenoids concentrations in the extracts and in the starting dry biomass. Number of replicates = 3.

	**Carotenoid**	**mg _**carotenoid**_/g _**extract**_**	**mg _**carotenoid**_/kg _**dry biomass**_**	**Relative percentages (%)**
sc-CO_2_	β-carotene	0.18 ± 0.03	0.67 ± 0.06	11 ± 1
	Echinenone	1.26 ± 0.05	4.6 ± 0.4	75 ± 6
	Astaxanthin	0.25 ± 0.01	0.92 ± 0.09	15 ± 1
sc-CO_2_EtOH	β-carotene	0.35 ± 0.05	2.0 ± 0.5	7 ± 1
	Echinenone	1.89 ± 0.08	11 ± 2	39 ± 4
	Astaxanthin	2.64 ± 0.09	15± 1	54 ± 7

From the results displayed in Table 2, the amount of astaxanthin is shown to 10-fold increase when ethanol was employed as co-solvent during the supercritical CO_2_ extraction. A slight increase (about 2-fold) was recorded also for echinenone and β-carotene.

### PHNQ Extraction

#### Solvent-Based Extraction

**Figure 4:**
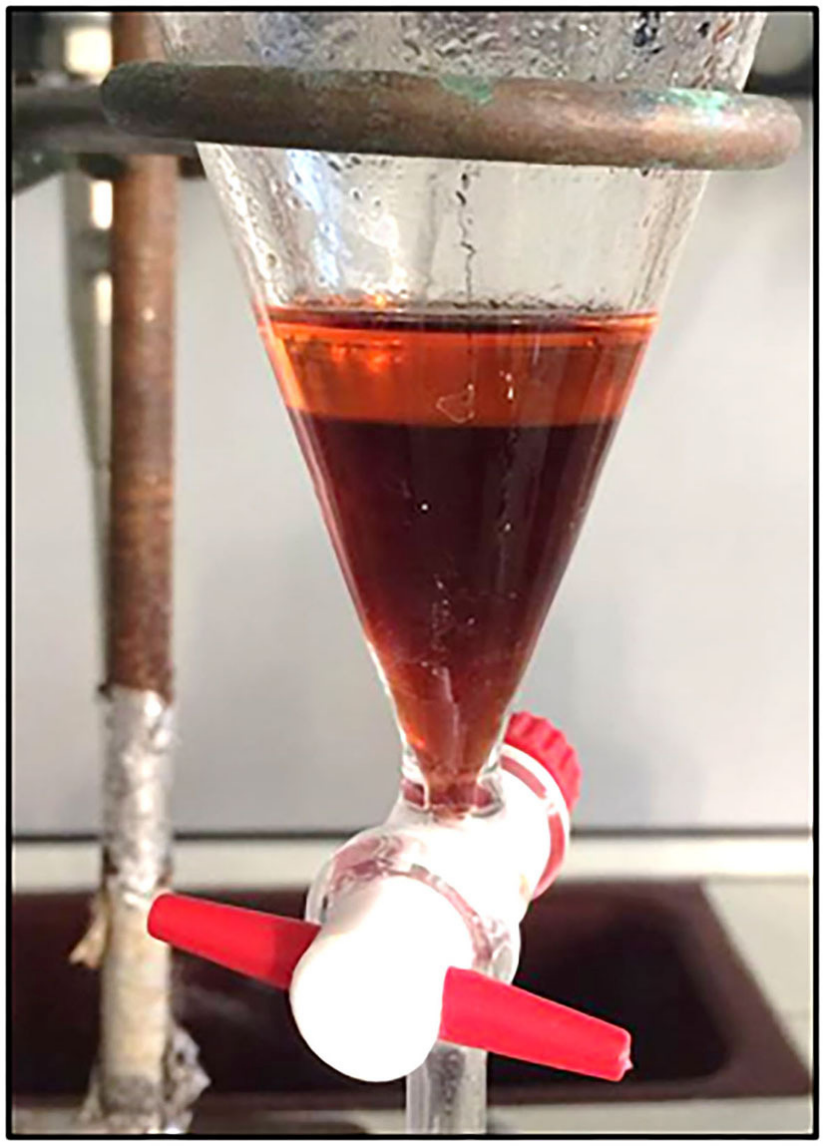
Separating funnel with phase separation during the counter-extraction. Lower phase: aqueous phase; upper phase: ethyl acetate phase containing polyhydroxynapthoquinones (PHNQ).

Attempts of extraction of PHNQ in supercritical conditions were carried out changing different physicochemical parameters and the co-solvent nature and percentages but none of the trials were found successful. For this reason, a modification of solvent-based extraction procedures was optimized. The starting carbonate matrix decomposition by aqueous formic acid is necessary to make the successive PHNQ extraction easier. Direct treatment of the powder of sea urchins with ethyl acetate is indeed unable to yield PHNQ. After formic acid decomposition and ethyl acetate counter extraction, the organic phase was characterized by a pink-orange color (as shown in the upper phase in Figure 4). Multiple washing steps with water were necessary to remove salts (calcium and magnesium formiate) due to the slight solubility of water in ethyl acetate that could affect the final PHNQ yield. The final yield was 0.07 ± 0.01% (on dry biomass). The extraction was run five times to get a standard deviation.

#### Total Phenolic Content

Total phenolic content was determined using the Folin Ciocalteu colorimetric assay. The extract displayed a high TPC value, expressed as gallic acid equivalents per gram of extract, corresponding to 441 ± 24 mg _GAE_/g _extract_.

#### PHNQ Extract Characterization

**Figure 5:**
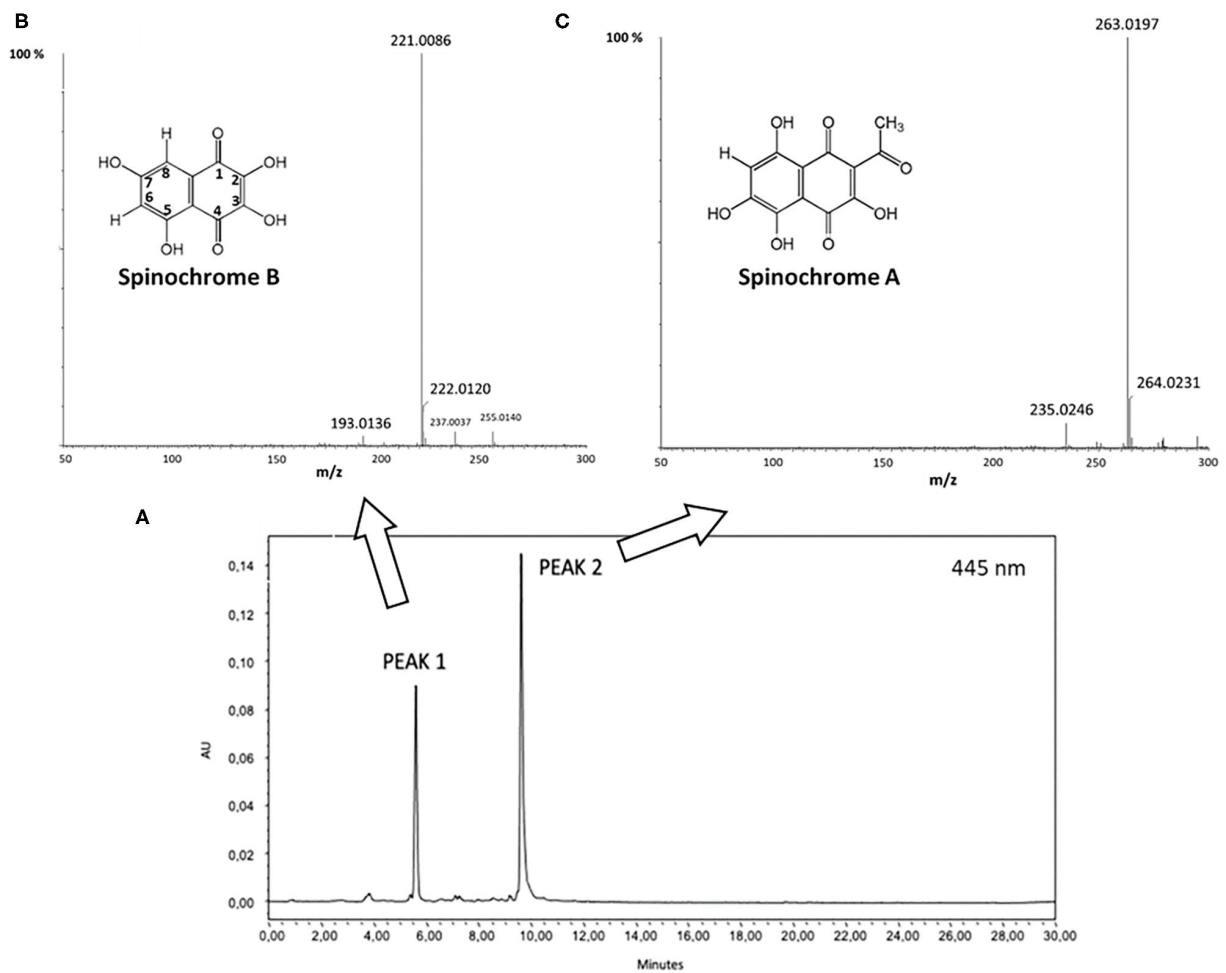
**(A)** Ultrahigh performance liquid chromatography-photodiode array (UPLC-PDA) chromatogram recorded at 445 nm; **(B)** Electrospray ionization-high resolution mass spectrometry (ESI-HRMS) relative to peak 1 (inset: molecular structure of Spinochrome B); **(C)** ESI-HRMS spectrum of peak 2 (inset: molecular structure of Spinochrome A).

After solvent removal by the evaporation under vacuum, the organic phase was injected in UPLC-PDA-ESI-HRMS to identify the PHNQ present in the extract. Figure 5 displays the results.

By *m/z* results, displayed in Figures 5B,C, Peak 1 and Peak 2 have been clearly attributed to Spinochrome B (*m/z* = 221.0086 Da; calculated mass = 221.0086 Da for C_10_H_5_O_6_) and Spinochrome A (*m/z* = 263.0197 Da; calculated mass = 263.0192 Da for C_12_H_7_O_7_) (12, 16).

#### PHNQ Antioxidant Activity

**Figure 6:**
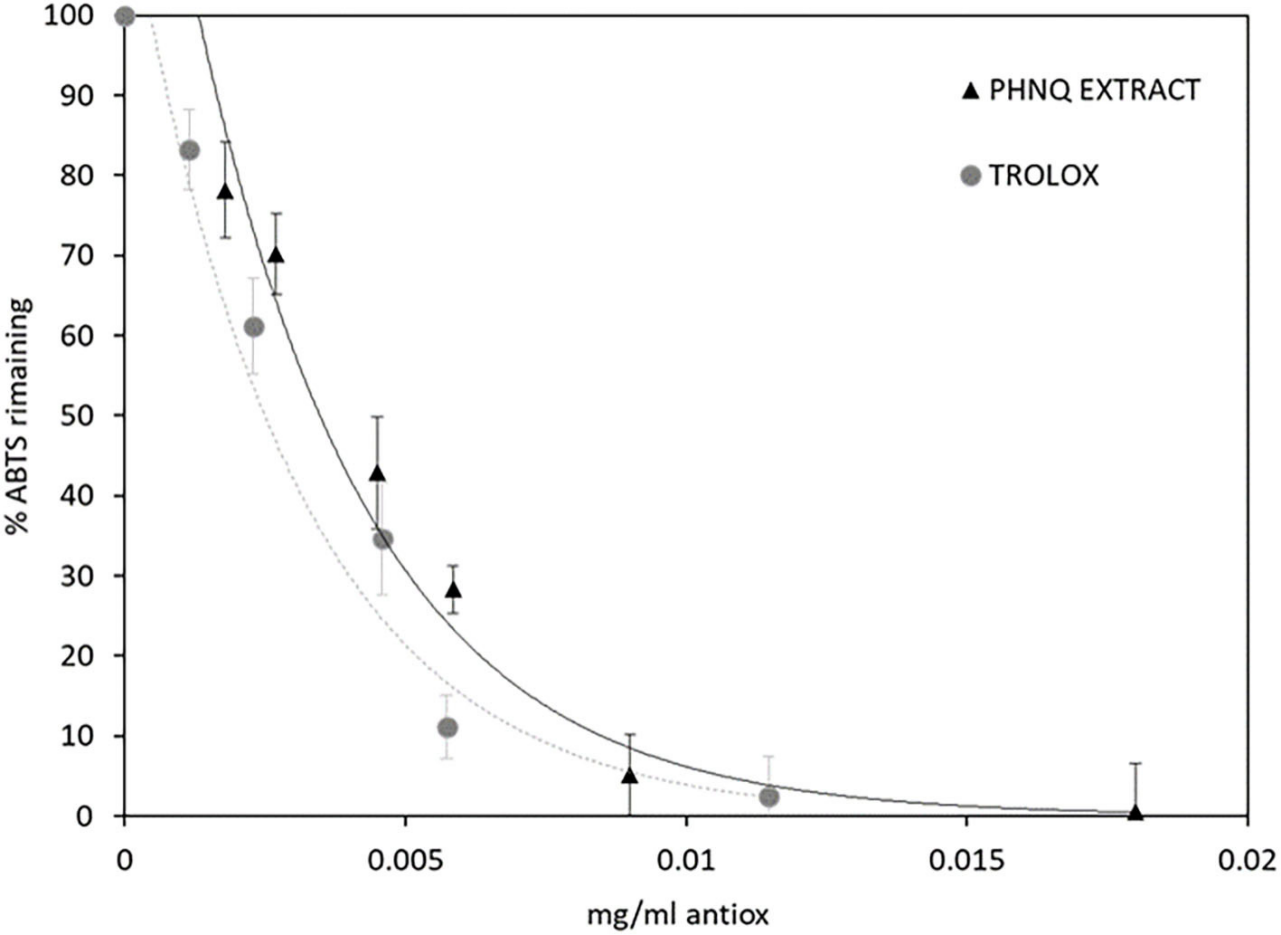
ABTS % remaining after the incubation period in the presence of different concentrations of PHNQ extract and Trolox®, as reference. Number of replicates = 3.

The radical scavenging activity of the PHNQ extract was determined by the ABTS assay and then compared to the water-soluble analog of vitamin E, Trolox®, and results are expressed as Trolox® equivalents. The radical scavenging activity of PHNQ extract was determined by the ABTS radical cation decolorization assay, as shown in Figure 6.

The EC_50_ values, calculated for both PHNQ extract and Trolox®, confirming the high antioxidant activity of the extract from the sea urchins, correspond to 0.004 ± 0.001 and 0.0035 ± 0.0005 mg ml^−1^, respectively. In terms of Trolox® equivalents, this value amounts to 875 ± 252 mg _Trolox_ g ${}_{\text{extract}}^{-1}$.

## Discussion

### Lipids

The use of supercritical CO_2_ was found mandatory when targeting lipids from the waste of sea urchins. Probably due to matrix effects, mass transfer of compounds of interests was hindered when “conventional” organic solvents were employed (such as hexane, dichloromethane, and chloroform).

Both the initial extractions, carried out by using supercritical CO_2_ in the presence and absence of co-solvent, are characterized by similarities in their kinetics. Indeed, according to the literature, extraction curves can be divided into three kinetic stages, each one controlled by different mass transfer mechanisms: (1) the constant extraction rate (CER), where the convection is the dominant mass transfer mechanism; (2) the falling extraction rate (FER), where the diffusion mechanism starts, operating combined with convection; (3) the diffusion-controlled period, where the mass transfer occurs mainly by diffusion in the bed and inside the solid substrate particles (30). Figures 1A,B clearly displays these three steps, corresponding to a starting high rate of extraction, followed by the plateau, assessing and confirming the consecutive kinetics of extraction from the biomass. Different from the extraction performed in the presence of ethanol as co-solvent, when CO_2_ was present alone, the first stage of the extraction was steep (up to 200 L of employed CO_2_), confirming the prompt solubilization in the supercritical fluid of the extractable compounds. The steepness of the first stage of the extraction was, on the other hand, lower in the presence of ethanol as co-solvent, confirming a more hindered mass transfer of the extractable compounds in these physicochemical conditions.

The sc-CO_2_ extract (168 ± 10 mg), obtained from the pristine waste of sea urchin lyophilized powder at 60°C and 200 bar, containing about 41.6% of saturated fatty acids (SFA) and 56.2% of polyunsaturated fatty acids (PUFA). The ratio SFA/PUFA is in favor of the polyunsaturated fatty acids. Myristic, palmitic, and stearic acids were the main SFA (Table 1). The composition of long-chain polyunsaturated fatty acids (LC-PUFAs) was characterized by three major components, arachidonic acid in the largest amount, eicosapentaenoic acid, and eicosatrienoic acid with comparable relative concentrations. ω6 PUFA was indeed the main component, compared to ω3 and ω9. These LC-PUFAs possess important properties since they act as the precursors of eicosanoids, a family of bioactive lipid mediators that regulate a wide variety of physiological as well as pathological responses and often exhibit potent inflammatory properties (29).

The presence of cholesterol in the pure sc-CO_2_ extract is not surprising, being the main sterol among the constituents of the gonads of the sea urchins (10, 31).

Together with fatty acids and sterols, supercritical CO_2_ alone was also able to yield carotenoids.

In particular, as shown in Table 2, the most abundant carotenoid in the extract, as detected by UPLC, was echinenone (with a relative percentage of 75%). In particular, echinenone has been already studied in the literature as the major carotenoid found in the gonads of sea urchins (up to 50–60% of the total pigments) (32). This suggested that it may have a role in their reproduction (33, 34). In terms of relative concentration in the extract, echinenone was followed by astaxanthin (15%) and then β-carotene (11%). When the extraction was run on the previously extracted biomass, but in the presence of ethanol as co-solvent, acting as polarity modifier, 81 ± 5 mg of the extract have been collected.

In this extract, the relative percentages of detected carotenoids were deeply affected, now yielding astaxanthin as the major carotenoid in the sc-CO_2_EtOH extract (relative percentage of 54%). Bearing hydroxyl groups on the terminal ring systems of the carotenoid-based structure, astaxanthin is characterized by an enhanced polarity and hence is more easily extractable in the presence of a co-solvent. The amount of β-carotene slightly decreased and echinenone halves its presence down to 39%.

Looking at the concentrations of each carotenoid in the starting dry biomass (fourth column in Table 2), even if the values seem low in terms of carotenoids content (in the order of magnitude of mg per kilogram of dry biomass), these are in agreement with the literature data quantifying the amount of carotenoids from different sources such as feed additives for fishes and laying hens (35, 36). These additives are employed in this field because carotenoids, such as astaxanthin, are demonstrated to promote growth rates during the early start-feeding period. Subsequently, positive effects of carotenoids as supplements, even in the order of mg per kg of diet, to commercial start-feeding diets, on the growth of some fish species, have been reported.

### Polyhydroxynapthoquinones

As a further step, after carotenoids extraction, identification, and quantification, the focus was shifted to the PHNQ class of compounds. Due to their polyhydroxylated quinonoid nature, their extractability in supercritical conditions is hindered, even in the presence of different co-solvents (ethanol, methanol, water, etc.). After different experimental trials, a solvent-based extraction was assessed as the optimal strategy, performed with modifications with respect to some literature methods (16). The main constituents of the wastes of sea urchins are calcium and magnesium carbonate, accounting for more than 90% of their weight, PHNQs are hence compartmented inside the inorganic matrix (37). By ICP-OES analyses, the molar ratio between Ca and Mg was found to be 16:1. When formic acid decomposes the carbonate-based powder of sea urchins, the following reaction occurs, yielding calcium formiate (an analog reaction occurs for magnesium, producing magnesium formiate):


(2) $$\begin{array}{r} {\text{Ca}}^{2+}+{\text{CO}}_{3}^{2-}+2\text{HCOOH}\to {\text{Ca}}^{2+}+2{\text{HCOO}}^{-}+{\text{CO}}_{2}+{\text{H}}_{2}\text{O} \end{array}$$

In addition, Ca and Mg formiate, highly soluble in water, can partially contaminate the ethyl acetate phase, affecting the PHNQ yield. This is due to the partial solubility of water in ethyl acetate (3.3% at 20°C) (38). For this reason, many washing steps by water aliquots were found necessary to remove salts from the ethyl acetate phase containing PHNQ. The specific conductivity of each successive aliquot of water was measured until its value was comparable to the pristine deionized water. The final yield, equal to 0.07 ± 0.01% by weight of the starting biomass, was hence not affected by the presence of residual salts.

The presence of phenolic structures in the obtained extract was confirmed by Folin–Ciocateu assay. In the absence of authentic standards, not commercially available, the determination of the TPC is a good measure of the presence of the targeted compounds. In terms of gallic acid equivalents, the TPC (441 ± 24 mg _GAE_/g _extract_) was surprisingly high, confirming the successful extraction of PHNQ and salts removal.

The identification of Spinochrome B and Spinochrome A by UPLC-HRMS was in agreement with the literature data, since the species under study, *Paracentrotus lividus*, has been already demonstrated to contain these pigments (14, 39).

Molar extinction coefficients of spinochrome A (ε = 3,311 l mol^−1^ cm^−1^ at 520 nm) and spinochrome B (ε = 4,898 mol^−1^ cm^−1^ at 480 nm) have been already determined in the literature and quantification of the content of each spinochrome A and B were performed by using Lawsone as standard, considering the relative ratio between its molar extinction coefficient (ε = 1,2000 L mol^−1^ cm^−1^ at 462 nm) and the PHNQ ones (40, 41). Lawsone, 2-hydroxy-1,4-naphthoquinone, is indeed structurally similar to PHNQ, bearing a monohydroxylated naphtoquinone skeleton. By this calculation, the extract was demonstrated to contain 19% by weight of Spinochrome B and 21% by weight of Spinochrome A, accounting for 40% of the weight of the extract. This value is in agreement with the TPC content previously determined.

The antioxidant properties of PHNQ pigments have been investigated in several different models. Interestingly, it was shown that the radical scavenging activity of pigments depends on the number and positions of the hydroxyl groups. In detail, the hydroxyl groups in the C-2, C-3, and C-7 positions (as shown numeration in Figure 5) play a beneficial role in the radical scavenging capacity, while the hydroxyl groups at C-5 and C-8 positions hinder hydrogen atom donation and adversely influence the free radical scavenging capacity (14). Spinochrome B displays all the OH beneficial groups in 2, 3, and 7 positions, Spinochrome A carries only one beneficial OH group in position 3 of the quinonoid ring. These features, together with their relative quantification in the extract, account for the high antioxidant activity measured by the ABTS radical cation decolorization assay. The EC_50_ value measured for the PHNQ extract, equal to 0.004 ± 0.001 mg ml^−1^, is comparable to the EC_50_ of Trolox® (0.0035 ± 0.0005 mg ml^−1^) and with results from Utkina et al. (42). Other studies in the literature found higher EC_50_ values, due to differences in the content of the pigment, depending on the investigated species of the sea urchin (43).

## Conclusion

This study validates the use of successive extraction strategies of added-value compounds, targeting specific chemical species, characterized by different polarities, aiming at valorizing the wastes of sea urchin. Sea urchin's waste whole content was indeed investigated for the first time in its lipidic species, belonging to the fatty acids and carotenoids classes, which were successfully extracted by means of a green technique, supercritical CO_2_ in the absence, and presence of a co-solvent like ethanol. PUFA and carotenoids such as echinenone, astaxanthin, and β-carotene were identified and quantified in the extracts. Their presence is possibly attributable to the residues of gonads and other soft tissue remains (e.g., digestive tract) after sea urchins consumption as a food delicacy. The defatted biomass, comprising the tests and spines, was then re-extracted to yield polyhydroxy-1,4-naphtoquinones, whose high interest is related to the multiple antioxidants, biological, and pharmacological activities.

Overall, the antioxidant activities measured for the PHNQ were highly remarkable, pointing to the use of this kind of waste for multiple applications when the radical scavenging activities are key features.

For example, they could be used as an additive in the further development of sea urchin collagen-based biomaterials to produce bioactive devices for regenerative medicine able to simultaneously provide both structural/filler (collagen) as well anti-oxidant and anti-inflammatory (PHNQ) properties.

The presence of added-value lipid compounds, such as PUFA and carotenoids, together with the more polar hydroxylated quinonoid-like compounds, Spinochrome B and Spinochrome A, enriching the carbonate-based matrix, opens the way to potential reuses of the waste toward other applications. The latter could be related, for example, to the development of feed additives for animals specifically requiring a high amount of calcium carbonates, such as laying hens or sea urchins themselves. Particularly, the development of optimal growth-promoting feed for sea urchin aquaculture (echinoculture) will hopefully promote this practice and reduce the impacts on natural stocks.

## Data Availability Statement

The raw data supporting the conclusions of this article will be made available by the authors, without undue reservation.

## Author Contributions

SM was responsible for study design, supervised experiments, and wrote and revised the manuscript. GM performed the experiments and wrote part of the manuscript. MS refined and revised the manuscript. LV supervised the project, refined, and revised the manuscript. All authors contributed to the article and approved the submitted version.

## Funding

The present research was financed by Cariplo Foundation (CIRCULAr project−2019-2169) and MIUR-PRIN (BRITEs project−2017FNZPNN).

## Conflict of Interest

The authors declare that the research was conducted in the absence of any commercial or financial relationships that could be construed as a potential conflict of interest.

## Publisher's Note

All claims expressed in this article are solely those of the authors and do not necessarily represent those of their affiliated organizations, or those of the publisher, the editors and the reviewers. Any product that may be evaluated in this article, or claim that may be made by its manufacturer, is not guaranteed or endorsed by the publisher.

## References

[1] United Nations. Department of Economic and Social Affairs Sustainable Development. Available online at: https://sdgs.un.org/goals (accessed June 14, 2021).

[2] Zilia F Bacenetti J Sugni M Matarazzo A Orsi L. From waste to product: circular economy applications from sea Urchin. Sustainability. (2021) 13:5427. 10.3390/su13105427

[3] Stefánsson G Kristinsson H Ziemer N Hannon C James P. Markets for Sea Urchins: A Review of Global Supply and Markets. Skýrsla Matís (2017) 45. p.

[4] Grisolía JM López F Ortúzar J de D. Sea urchin: from plague to market opportunity. Food Qual Prefer. (2012) 25:46–56. 10.1016/j.foodqual.2012.01.004

[5] Di Benedetto C Barbaglio A Martinello T Alongi V Fassini D Cullorà E. Production, characterization and biocompatibility of marine collagen matrices from an alternative and sustainable source: the sea urchin *Paracentrotus lividus*. Mar Drugs. (2014) 12:4912–33. 10.3390/md12094912 25255130 PMC4178497

[6] Ferrario C Rusconi F Pulaj A Macchi R Landini P Paroni M. From food waste to innovative biomaterial: sea urchin-derived collagen for applications in skin regenerative medicine. Mar Drugs. (2020) 18:414. 10.3390/md18080414 32781644 PMC7460064

[7] Ferrario C Leggio L Leone R Di Benedetto C Guidetti L Coccè V. Marine-derived collagen biomaterials from echinoderm connective tissues. Mar Environ Res. (2017) 128:46–57. 10.1016/j.marenvres.2016.03.007 27063846

[8] Melotti L Martinello T Perazzi A Iacopetti I Ferrario C Sugni M. A prototype skin substitute, made of recycled marine collagen, improves the skin regeneration of sheep. Animals. (2021) 11:1–20. 10.3390/ani11051219 33922557 PMC8145883

[9] Carroll AR Copp BR Davis RA Keyzers RA Prinsep MR. Marine natural products. Nat Prod Rep. (2020) 37:175–223. 10.1039/C9NP00069K 32025684

[10] Shikov AN Laakso I Pozharitskaya ON nen-Laakso TS Krishtopina AS Makarova MN. Chemical profiling and bioactivity of body wall lipids from strongylocentrotus droebachiensis. Mar Drugs. (2017) 15:1–11. 10.3390/md15120365 29186813 PMC5742825

[11] Brasseur L Hennebert E Fievez L Caulier G Bureau F Tafforeau L. The roles of spinochromes in four shallow water tropical sea urchins and their potential as bioactive pharmacological agents. Mar Drugs. (2017) 15:179. 10.3390/md15060179 28621734 PMC5484129

[12] Brasseur L Caulier G Flammang P Eeckhaut I Demeyer M Decroo C. Identification and quantification of spinochromes in body compartments of *Echinometra mathaei's* coloured types. R Soc Open Sci. (2018) 5:171213. 10.1098/rsos.171213 30224975 PMC6124065

[13] Vasileva E Mishchenko NP. Diversity of polyhydroxynaphtoquinone pigments in north pacific sea urchins. Chem Biodivers. (2017) 14:e1700182. 10.1002/cbdv.201700182 28557305

[14] Shikov AN Pozharitskaya ON Krishtopina AS Makarov VG. Naphthoquinone pigments from sea urchins: chemistry and pharmacology. Phytochem Rev. (2018) 17:509–34. 10.1007/s11101-018-9547-3

[15] Rubilar T Barbieri ES Gazquez A Avaro M. Sea urchin pigments: echinochrome a and its potential implication in the cytokine storm syndrome. Mar Drugs. (2021) 19:267. 10.3390/md19050267 34064550 PMC8151293

[16] Powell C Hughes AD Kelly MS Conner S Mc Dougall GJ. Extraction and identification of antioxidant polyhydroxynaphthoquinone pigments from the sea urchin, *Psammechinus miliaris*. LWT - Food Sci Technol. (2014) 59:455–60. 10.1016/j.lwt.2014.05.016

[17] Slinkard K Singleton VL. Total phenol analysis: automation ans comparison with manual methods. Am J Enol Vitic. (1977) 28:49–55.

[18] Loganayaki N Siddhuraju P Manian S. Antioxidant activity and free radical scavenging capacity of phenolic extracts from *Helicteres isora L*. and *Ceiba pentandra L. J Food Sci Technol*. (2013) 50:687–95. 10.1007/s13197-011-0389-x 24425970 PMC3671060

[19] Tripodi F Lombardi L Guzzetti L Panzeri D Milanesi R Leri M. Protective effect of Vigna unguiculata extract against aging and neurodegeneration. Aging. (2020) 12:19785–803. 10.18632/aging.104069 33024055 PMC7732273

[20] Ermer J. Validation in pharmaceutical analysis. Part I: an integrated approach. J Pharm Biomed Anal. (2001) 24:755–67. 10.1016/s0731-7085(00)00530-6 11248468

[21] Marzorati S Schievano A Idà A Verotta L. Carotenoids, chlorophylls and phycocyanin from Spirulina: supercritical CO_2_ and water extraction methods for added value products cascade. Green Chem. (2020) 22:187–96. 10.1039/c9gc03292d

[22] Miao F Lu D Li Y Zeng M. Characterization of astaxanthin esters in *Haematococcus pluvialis* by liquid chromatography-atmospheric pressure chemical ionization mass spectrometry. Anal Biochem. (2006) 352:176–81. 10.1016/j.ab.2006.03.006 16597431

[23] Jonathan Posner Bradley S. Peterson JAR. Atmospheric pressure chemical ionization tandem mass spectrometry of carotenoids. Int J Mass Spectrom. (2012) 312:163–72. 10.1016/j.ijms.2011.07.030 22408388 PMC3293484

[24] Rivera SM Christou P Canela-Garayoa R. Identification of carotenoids using mass spectrometry. Mass Spectrom Rev. (2014) 33:353–72. 10.1002/mas.21390 24178708

[25] Li H Tyndale ST Heath DD Letcher RJ. Determination of carotenoids and all-trans-retinol in fish eggs by liquid chromatography-electrospray ionization-tandem mass spectrometry. J Chromatogr B Anal Technol Biomed Life Sci. (2005) 816:49–56. 10.1016/j.jchromb.2004.11.005 15664333

[26] Chakrabarti R. Carotenoprotein from tropical brown shrimp shell waste by enzymatic process. Food Biotechnol. (2002) 16:81–90. 10.1081/FBT-120004202

[27] Rivera S Vilaró F Canela R. Determination of carotenoids by liquid chromatography/mass spectrometry: effect of several dopants. Anal Bioanal Chem. (2011) 400:1339–46. 10.1007/s00216-011-4825-6 21380750

[28] Davies BH. Analysis of Carotenoid Pigments in Chemistry Biochemistry of Plant Pigment. ed Goodw TW. London, New York, NY: Academic Press (1965).

[29] Tomas RN Cox ER Steidinger KA. Pigments of the dinoflagellate peridinium balticum and its photosynthetic endosymbiont. J Phycol. (1977) 13:354–8.

[30] Nasti R Galeazzi A Marzorati S Zaccheria F Ravasio N Luisa G. Valorisation of coffee roasting by - products : recovery of silverskin fat by supercritical - CO_2_ extraction. Waste Biomass Valoriz. (2021). 10.1007/s12649-021-01435-9

[31] López-Hernández J González-Castro MJ Piñeiro-Sotelo M. Determination of sterols in sea urchin gonads by high-performance liquid chromatography with ultraviolet detection. J Chromatogr Sci. (1999) 37:237–9. 10.1093/chromsci/37.7.237

[32] Symonds RC Kelly MS Caris-Veyrat C Young AJ. Carotenoids in the sea urchin *Paracentrotus lividus*: occurrence of 9′-cis-echinenone as the dominant carotenoid in gonad colour determination. Comp Biochem Physiol - B Biochem Mol Biol. (2007) 148:432–44. 10.1016/j.cbpb.2007.07.012 17765578

[33] Galasso C Corinaldesi C Sansone C. Carotenoids from marine organisms: biological functions and industrial applications. Antioxidants. (2017) 6:96. 10.3390/antiox6040096 29168774 PMC5745506

[34] Tsushlma M Kawakami M Mine M Matsuno T. The role of carotenoids in the development of the sea urchin pseudocentrotus depressus. Invertebr Reprod Dev. (1997) 32:149–53. 10.1080/07924259.1997.9672616

[35] Moreno JA Díaz-Gómez J Nogareda C Angulo E Sandmann G Portero-Otin M. The distribution of carotenoids in hens fed on biofortified maize is influenced by feed composition, absorption, resource allocation and storage. Sci Rep. (2016) 6:35346. 10.1038/srep35346 27739479 PMC5064355

[36] Meyers SP. Developments in world aquaculture, feed formulations, and role of carotenoids. Pure Appl Chem. (1994) 66:1069–76. 10.1351/pac199466051069

[37] Drozdov AL Sharmankina VV Zemnukhova LA Polyakova NV. Chemical composition of spines and tests of sea urchins. Biol Bull. (2017) 43:521–31. 10.1134/S106235901606007832759777

[38] Ethyl Acetate Solvent Properties. Available online at: https://macro.lsu.edu/HowTo/solvents/ethylacetate.htm (accessed June 9, 2021).

[39] Goodwin TW Srisukh S. A study of the pigments of the sea-urchins, *Echinus esculentus* L. and Paracentrotus lividus Lamarck. Biochem J. (2015) 47:69–76. 10.1042/bj0470069 14791308 PMC1275162

[40] Soleimani S Yousefzadi M moein S Rezadoost H Bioki NA. Identification and antioxidant of polyhydroxylated naphthoquinone pigments from sea urchin pigments of *Echinometra mathaei*. Med Chem Res. (2016) 25:1476–83. 10.1007/s00044-016-1586-y

[41] Khadtare SS Ware AP Salunke-Gawali S Jadkar SR Pingale SS PathanShubhangi HM. Dye sensitized solar cell with lawsone dye using ZnO photoanode: experimental and TD-DFT study. RSC Adv. (2015) 5:17647–52. 10.1039/C4RA14620D

[42] Utkina NK Pokhilo ND. Free radical scavenging activities of naturally occurring and synthetic analogues of sea urchin naphthazarin pigments. Nat Prod Commun. (2012) 7:901–4. 10.1177/1934578X1200700725 22908577

[43] Pastrana-Franco OJ Santafé-Patiño GG Quirós-Rodríguez JA. Antioxidant activity of the sea urchin *Mellita quinquiesperforata* (Leske) and identification of its major lipids compounds. Actual Biol. (2016) 38:14–22. 10.17533/udea.acbi.v38n104a02

